# Asmeton minimizes dry cough and diaphragmatic contraction during pulsed-field ablation of atrial fibrillation: a clinical prospective randomized study

**DOI:** 10.3389/fphys.2025.1702254

**Published:** 2026-02-18

**Authors:** Huixing Liu, Yan Zhang, Huafen Liu, Wei Liu, Zhuo Wang, Can Leng, Gangjie Xie, Jiahao Zhao, Jinlin Huang, Lei Huang, Yuzhi Lu, Hongwei Zhang, Songyun Wang, Hong Jiang

**Affiliations:** 1 Department of Cardiology, Renmin Hospital of Wuhan University, Wuhan, China; 2 Cardiac Autonomic Nervous System Research Center of Wuhan University, Wuhan, China; 3 Cardiovascular Research Institute, Wuhan University, Wuhan, China; 4 Hubei Key Laboratory of Cardiology, Wuhan, China; 5 Department of Cardiology, Enshi Huiyi Hospital of Rheumatic Diseases, Enshi, China

**Keywords:** asmeton, atrial fibrillation, diaphragmatic contraction, dry cough, pulsed-field ablation

## Abstract

**Purpose:**

Pulsed-field ablation (PFA) is a novel ablation modality with promising outcomes for atrial fibrillation therapy. However, PFA might lead to dry cough and diaphragmatic contraction, especially under conscious sedation. We aim to explore a novel approach to reduce dry cough and diaphragmatic contraction during PFA procedures performed under conscious sedation.

**Methods:**

A total of 16 patients who underwent PFA pulmonary vein isolation under conscious sedation were divided into the asmeton (with preprocedure asmeton) and control groups. A scoring system was developed to assess dry cough and diaphragmatic contraction.

**Results:**

A total of 608 ablations and 893 ablations were performed in the control and asmeton groups, respectively. The dry cough score (*P* = 0.045) in all pulmonary veins was significantly reduced by 73.1% in the asmeton group in comparison with the control group. The proportion of medium-to-high dry cough incidences decreased from 12.2 ± 10.4% in the control group to 1.8 ± 3.7% in the asmeton group (*P* = 0.027). The diaphragmatic contraction score in the asmeton group was 37.5% lower than that in the control group, and the proportion of severe diaphragmatic contraction incidences was significantly reduced from 2.8 ± 8.4% in the control group to 0.0 ± 0.0% in the asmeton group (*P* = 0.006).

**Conclusion:**

Asmeton might eliminate moderate-to-severe dry cough and reduce the severity of diaphragmatic contraction during pulmonary vein isolation under conscious sedation ablation.

## Introduction

Atrial fibrillation (AF) is a common cardiac arrhythmia that considerably increases the risk of stroke, heart failure, and other complications, thereby severely affecting patients’ quality of life and prognosis. Catheter ablation is recommended as the first-line treatment for AF. Traditional techniques of catheter ablation mainly include radiofrequency (RF) and cryoballoon ablation, but these thermal ablations result in serious complications and clinical adverse events (Calkins et al., 2017). Pulsed-field ablation (PFA) is an emerging non-thermal ablation modality for AF patients that compared to the traditional thermal ablation methods might lead to cell death in a more targeted and potentially safer manner with lower complication rate (Maor et al., 2019; Sugrue et al., 2019; Qiu et al., 2022; Reddy et al., 2018; Turagam et al., 2023; Reddy et al., 2023).

In the United Kingdom and other countries, the combination of benzodiazepines and opioids with conscious sedation is commonly used for thermal ablation (Chu et al., 2023; Calvert et al., 2024a). Although few previous studies have tested the feasibility of PFA under conscious sedation, general anesthesia is commonly seen in PFA (Reddy et al., 2019) because of the side effects, such as diaphragm contraction and dry cough (Maor et al., 2019; Sugrue et al., 2019; Reddy et al., 2019; Schmidt et al., 2022; Ekanem et al., 2022). These side effects may cause discomfort to patients and impact the efficiency and safety of the treatment. Previous studies have shown that intraoperative breath control might reduce the impact of PFA on diaphragmatic contraction and dry cough under conscious sedation (Jiang et al., 2023). However, performing respiratory control during the operation is complicated, requires the cooperation of the patient and the operator, and even requires practice, which greatly prolongs the duration and poses a risk of improper operation. Therefore, exploring a novel approach to reduce these side effects, for instance, through the use of certain medications, might be an important area of research.

Asmeton is a common and effective cough medicine that might relieve dry cough owing to different mechanisms, such as relaxing bronchial smooth muscle, preventing the introduction of cough impulses, and preventing allergic reactions in the respiratory mucosa. This study aims to explore whether asmeton could minimize the degree of diaphragmatic contraction and dry cough caused by PFA during pulmonary vein isolation (PVI).

## Methods

### Study design and patient population

The clinical trial, a prospective study involving 16 patients conducted to evaluate the safety and effectiveness of the Pulstamper PFA catheter (APT Medical Inc., China) for treating symptomatic AF, was approved by the participating medical center’s ethics committee of Renmin Hospital of Wuhan University (WDRY2021-K111). Detailed patient inclusion and exclusion criteria are listed in the Supplementary Material (Supplementary Table S1). Patients were randomly divided into the asmeton and the control groups after enrollment.

### Investigational device

The Pulstamper PFA catheter is used to treat AF by inducing irreversible electroporation in atrial myocardiocytes and electrically isolating PVs in the left atrium (LA). The Pulstamper PFA catheter has a distal circular shape and consists of seven ring electrodes. All electrodes are capable of recording intracardiac electrograms using a 3D cardiac mapping system. Each ablation involves a series of five discharges. All patients participated this study after signing an informed consent statement for the pulsed-field ablation procedure. Pre-procedural screening including the medical history, blood testing, left atrial pulmonary vein computed tomography angiography (CTA), and trans-esophageal echocardiography (TEE) of the heart was conducted to ensure that all patients met the inclusion criteria. All procedures were performed under conscious sedation with intravenous administration of fentanyl (240 μg/h), and patients were able to respond to audible instructions from physicians. For the procedure, after femoral venous access, a 6-F decapolar diagnostic catheter was placed in the coronary sinus. Under fluoroscopic guidance, trans-septal puncture was performed using an 8.5-F steerable sheath (APT Medical Inc., China), following which a PFA catheter was guided into the LA until it reached the desired location at the ostium of each PV. Anatomical models and bipolar voltage maps of the LA were created using a Pulstamper catheter in the 3D cardiac mapping system (HT Viewer® Magnetoelectric positioning 3D mapping system). Pulsed-field ablation energy was applied to form a contiguous lesion to isolate PVs. The number of ablations in which the potential disappeared and the total number of ablations were recorded.

### Rating scores

Immediately after surgery, patients were evaluated for intraoperative feelings and symptoms of intraoperative chest pain, chest tightness, headache, and head swelling. The scoring table for each symptom is shown in Supplementary Table S1. The overall score ranged from 0 to 16. Scores were rated from 0 (no response) to 16 (strong response). For each ablation, trained independent technicians observed and scored the PFA-stimulated dry cough and diaphragmatic contraction (Jiang et al., 2023) using the scoring system described in Supplementary Tables S2 and S3. Scores were rated from 0 (no response) to 3 (high response). Scores were rated by the same technician for each patient to ensure data consistency.

### ELISA

Peripheral venous blood samples were collected from each patient at two time points: (1) preoperatively (before the ablation procedure) and (2) immediately postoperatively.

The blood sample is placed at room temperature for 2 h or 4 °C overnight and then centrifuged at 2 °C–8 °C at 3,000 rpm for 15 min, and the supernatant can be detected immediately. The antibody is then diluted with carbonate coating buffer to 1 μg/mL–10 μg/mL protein content. Then, after blocking, the antibody is added, and finally, enzyme conjugates are added. The standard curve was made according to the concentration and O.D. of the standard, and then the sample concentration was calculated according to the standard curve equation. The ELISA experiment was completed using the equipment provided by Wuhan Servicebio Technology Co., Ltd.

### Statistical analysis

This study focused on the quantitative analysis of a method to reduce the impact of PFA on diaphragm movement and coughing. For analyzing patient baseline characteristics and procedural parameters, the number, mean, standard deviation (SD), and included 95% confidence intervals were included. Normality tests were performed before other analyses to assess if the scores followed a normal (Gaussian) distribution. If the scores did not follow a normal distribution, a non-parametric unpaired t-test was used to compare the difference between the scores of the control and test ablations. Fisher’s exact test was used to compare the proportion and incidence rate between the control and asmeton groups. The difference was considered significant if P < 0.05. All statistical analyses were performed using GraphPad Prism version 9.3.1 (San Diego, CA, United States) and SPSS 25.

## Results

### Patient baseline characteristics

The study (Table 1) included 16 patients randomly divided into the control group (n = 7) and the asmeton group (n = 9), and the baseline characteristics of these two groups were compared. The results indicated that there were no statistically significant differences between these two groups in terms of age, gender, BMI, disease duration, average heart rate, cardiac structure and function indicators, and anticoagulant use, indicating that these two groups were highly comparable in most baseline characteristics.

**TABLE 1:** Baseline patient characteristics (N = 16).

Characteristics	Control group (n = 7)	Asmeton group (n = 9)	P-value
Age, years	63.7 ± 6.9	66.7 ± 11.1	0.548
Male, n (%)	3 (42.9%)	5 (55.6%)	0.642
Percentage of the first ablation, n (%)	5 (71.4%)	7 (77.8%)	0.789
BMI	23.5 ± 1.2	25.6 ± 3.8	0.219
Course of disease, months	17.8 ± 45.1	16.2 ± 22.5	0.927
Average heart rate, beats per minute	70.4 ± 9.3	84.7 ± 27.3	0.210
LA diameter, mm	39.9 ± 3.0	41.1 ± 5.1	0.574
LVEF, %	60.0 ± 0.0	54.2 ± 7.7	0.068
LV diameter, mm	47.3 ± 5.4	54.2 ± 7.7	0.704
Warfarin	0 (0%)	0 (0%)	1.000
NOAC	7 (100%)	9 (100%)	1.000

Values are presented as the mean ± SD or n (%).

BMI, body mass index; LA, left atrium; LVEF, left ventricular ejection fraction; LV, left ventricular; NOAC, non-vitamin K oral anticoagulants.

### Procedural and ablation characteristics

This study (Table 2) compared the procedural and ablation characteristics between the control group (n = 7) and the asmeton group (n = 9). Acute PVI was achieved in 100% of patients in both groups, with no significant difference (*P* = 1.000). Most procedural metrics, including changes in diaphragmatic excursion, activated coagulation time, fluoroscopy time, total procedure time, and ablation-related parameters (ablation time and number of lesions), did not have significant differences between the groups. No adverse event (AE) or serious adverse event (SAE) occurred in either group (*P* = 1.000). Overall, most procedural and safety parameters were similar. When comparing the left and right pulmonary veins, no significant difference was found in the impedance (*P* = 0.64), total number of ablations (*P* = 0.29) and ablation time (*P* = 0.82), as shown in Table 3.

**TABLE 2:** Procedural and ablation characteristics (N = 16).

Procedural and ablation characteristics	Control group (n = 7)	Asmeton group (n = 9)	P value
Successful acute PVI, %	100%	100%	1.000
Fluoroscopy time, min	4.8 ± 1.2	5.8 ± 1.3	0.179
Procedure time (including the 30-min waiting period), min	106.8 ± 26.3	116.4 ± 24.7	0.485
Pre-ablation mapping time, min	11.0 ± 5.3	16.9 ± 10.5	0.231
LA dwell time, min	75.2 ± 29.6	91.4 ± 25.5	0.276
Ablation time to PVI, min	33.6 ± 17.9	43.8 ± 12.6	0.202
Total number of ablations	90.6 ± 43.6	116.7 ± 45.6	0.267
AE/SAE	0	0	1.000
Change of diaphragmatic excursion, mm	3.2 ± 7.2	−1.5 ± 5.4	0.365

Values are presented as the mean ± SD, median (interquartile range), or n (%).

PVI, pulmonary vein isolation; ACT, activated coagulation time; AE/SAE, adverse event/serious adverse event.

**TABLE 3:** Procedural and ablation characteristics of the left pulmonary vein and right pulmonary vein.

Items	Left pulmonary vein	Right pulmonary vein	P value
Impedance, Ω	121.7 ± 14.9	123.3 ± 15.0	0.64
Ablation time, min	18.4 ± 5.2	18.3 ± 7.6	0.82
Total number of ablations	44.9 ± 9.6	52.2 ± 26.7	0.29

### Ablation numbers and their distribution

**FIGURE 1:**
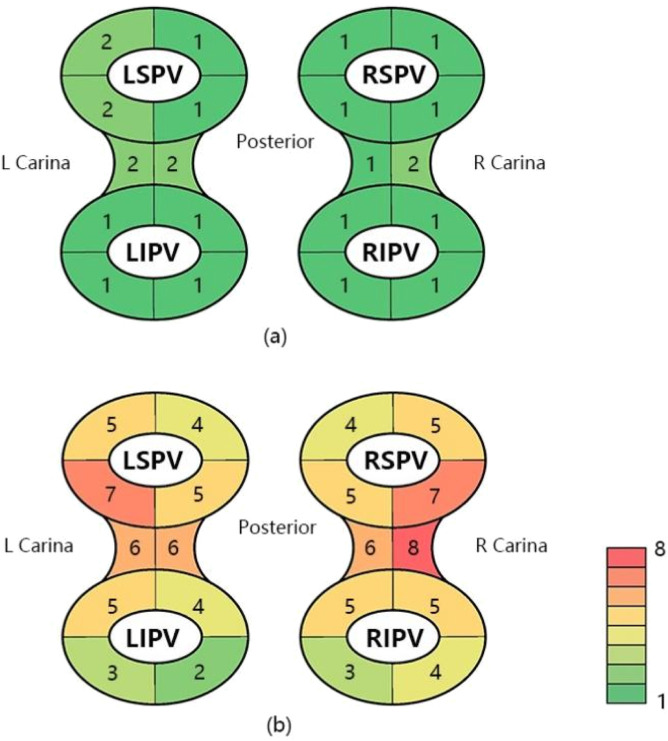
Number of potential loss ablations and total number of ablations for PVI. **(a)** Number of ablations at each site where the potential disappears during PVI. **(b)** Total number of ablations at each site during PVI. LSPV, left superior pulmonary vein; LIPV, left inferior pulmonary vein; RSPV, right superior pulmonary vein; RIPV, right inferior pulmonary vein.

In order to more intuitively demonstrate the difference in the relevant characteristics of different pulmonary veins during PFA, Figure 1 is plotted. The distribution varies among different PVs, but the overall trends can be observed. Except for the left superior pulmonary vein (LSPV) and the left and right carina, the remaining pulmonary vein potential disappeared after a single pulmonary vein ablation. To prevent pulmonary vein reconnection, consolidation ablation was performed, and of these, the left and right carina and pulmonary veins near carina require more ablation. In addition, the number of ablations of the anterior wall is higher than that of the posterior wall.

### Intraoperative feeling

**FIGURE 2:**
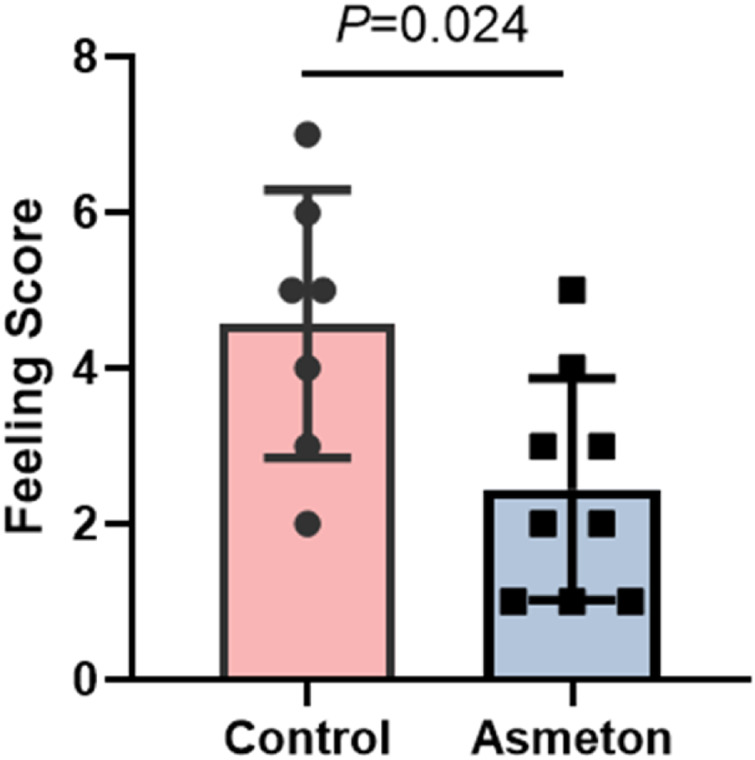
Intraoperative feeling score of the control group and asmeton group.

For the intraoperative feeling score, an average score of 3.4 ± 1.9 (no pain–most pain: 0–16) indicated acceptable feelings among patients during the operation, and after using asmeton, patients felt significantly better, with the intraoperative feeling score decreasing from 4.6 ± 1.7 to 2.4 ± 1.4 (Figure 2, *P* = 0.024).

### Dry cough score and diaphragm contraction

#### Dry cough

**FIGURE 3:**
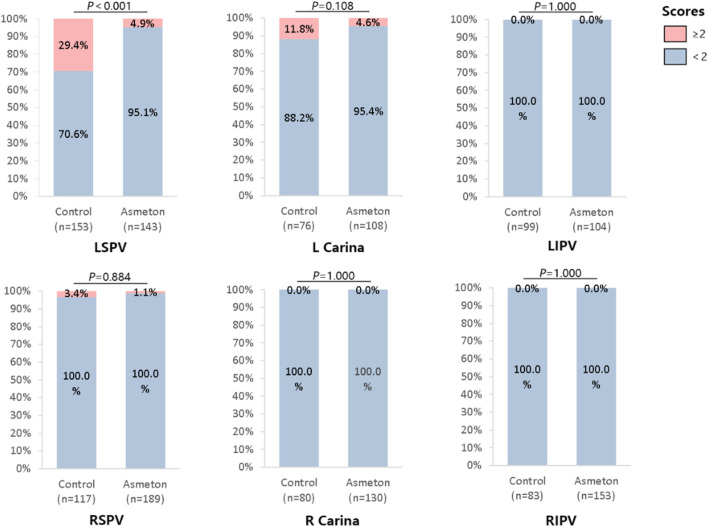
Distribution of scores of dry cough with or without asmeton in each PV. The proportion of ablations with a cough score of less than two points in different pulmonary veins with or without the use of asmeton. The proportion of ablations with a cough score of less than two points refers to the proportion of non-cough and mild cough cases in the total number of ablations. LSPV, left superior pulmonary vein; LIPV, left inferior pulmonary vein; RSPV, right superior pulmonary vein; RIPV, right inferior pulmonary vein.

The control and asmeton groups had 608 and 893 incidents of ablation, respectively, during PFA. The average score in the asmeton group (0.07 ± 0.07) was significantly lower than that in the Control group (0.26 ± 0.24, *P* = 0.045), with a reduction of 73.1%. Similar significant differences were also noted in some pulmonary veins, such as LSPV, where the score in the asmeton group (0.18 ± 0.22) was much lower than that in the control group (0.67 ± 0.59, *P* = 0.047), with a reduction of 73.1% (Table 4). This is consistent with the trend that asmeton might reduce the incidences of medium and high cough in LSPV (P < 0.001) (Figure 3). In general, the proportion with ≥2 cough scores decreased from 12.2% ± 10.4% to 1.8% ± 3.7% when using asmeton (*P* = 0.027). Figure 3 presents the distribution of scores of dry cough with or without asmeton in each PV. Cough is more likely to occur in LSPV, and less likely to occur in LIPV, R carina, and right inferior pulmonary vein (RIPV). The use of asmeton can significantly reduce the occurrence of cough during ablation in LSPV.

**TABLE 4:** Score of dry cough with or without asmeton during PFA in each PV.

Items	Control group (n = 608)	Asmeton group (n = 893)	P-value	Average score reduction (%)
Average score	0.26 ± 0.24	0.07 ± 0.07	0.045*	73.1%
LSPV	0.67 ± 0.59	0.18 ± 0.22	0.047*	73.1%
Left carina	0.29 ± 0.33	0.13 ± 0.19	0.346	55.2%
LIPV	0.07 ± 0.13	0 ± 0	0.118	100%
RSPV	0.07 ± 0.12	0.03 ± 0.05	0.592	57.1%
Right carina	0.08 ± 0.20	0.01 ± 0.02	0.751	87.5%
RIPV	0.02 ± 0.05	0.01 ± 0.04	0.642	50%

LSPV, left superior pulmonary vein; LIPV, left inferior pulmonary vein; RSPV, right superior pulmonary vein; RIPV, right inferior pulmonary vein. *P < 0.05.

#### Diaphragm contraction

**FIGURE 4:**
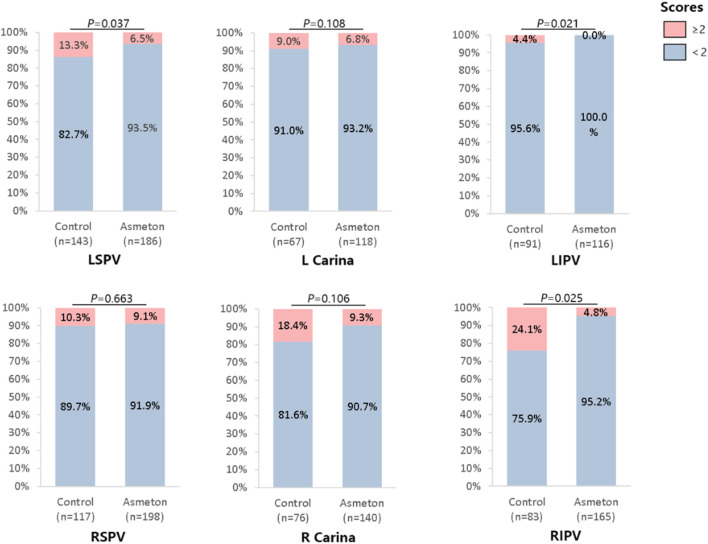
Distribution of scores of diaphragmatic contraction without Asmeton in each PV. This figure shows the proportion of ablations with a diaphragmatic contraction score of less than two points in different pulmonary veins with or without the use of asmeton. The proportion of ablations with a diaphragmatic contraction score of less than two points refers to the proportion of non-cough and mild diaphragmatic contraction in the total number of ablations. LSPV, left superior pulmonary vein; LIPV, left inferior pulmonary vein; RSPV, right superior pulmonary vein; RIPV, right inferior pulmonary vein.

In the study of diaphragm contraction during PFA with or without asmeton, the control and asmeton groups had 577 and 893 incidents of ablation, respectively. For diaphragmatic contraction, although the overall average score in the asmeton group decreased compared to that in the control group, the difference was not significant (*P* = 0.077). However, significant differences were observed in specific pulmonary veins. In the left inferior pulmonary vein (LIPV), the average score in the asmeton group (0.05 ± 0.10) was significantly lower than that in the control group (0.32 ± 0.23, P = 0.005), with a reduction of 84.4% (Table 5). In the distribution of scores, the proportion of high degree of diaphragmatic contraction decreased from 2.8% ± 3.3% in the control group to 0% ± 0% in the asmeton group (*P* = 0.006), with an average score reduction of 100%. Figure 4 shows the distribution of asmeton-group diaphragm contraction scores for each PV. The use of asmeton causes a reduction in medium-to-high diaphragm contraction, with significant differences in the LSPV (*P* = 0.037), LIPV (*P* = 0.021), and RIPV (*P* = 0.025).

**TABLE 5:** Score of diaphragm contraction with or without asmeton during PFA.

Items	Control group (n = 577)	Asmeton group (n = 893)	P-value	Average score reduction (%)
Average score	0.48 ± 0.25	0.30 ± 0.25	0.077	37.5%
LSPV	0.41 ± 0.42	0.30 ± 0.32	0.548	26.8%
Left carina	0.31 ± 0.28	0.29 ± 0.36	0.631	6.5%
LIPV	0.32 ± 0.23	0.05 ± 0.10	0.005*	84.4%
RSPV	0.37 ± 0.48	0.49 ± 0.50	0.787	−32.4%
Right carina	0.72 ± 0.94	0.37 ± 0.36	0.688	48.6%
RIPV	0.77 ± 0.51	0.31 ± 0.40	0.092	59.7%

LSPV, left superior pulmonary vein; LIPV, left inferior pulmonary vein; RSPV, right superior pulmonary vein; RIPV, right inferior pulmonary vein.

#### Serum IL1β and IL10

**FIGURE 5:**
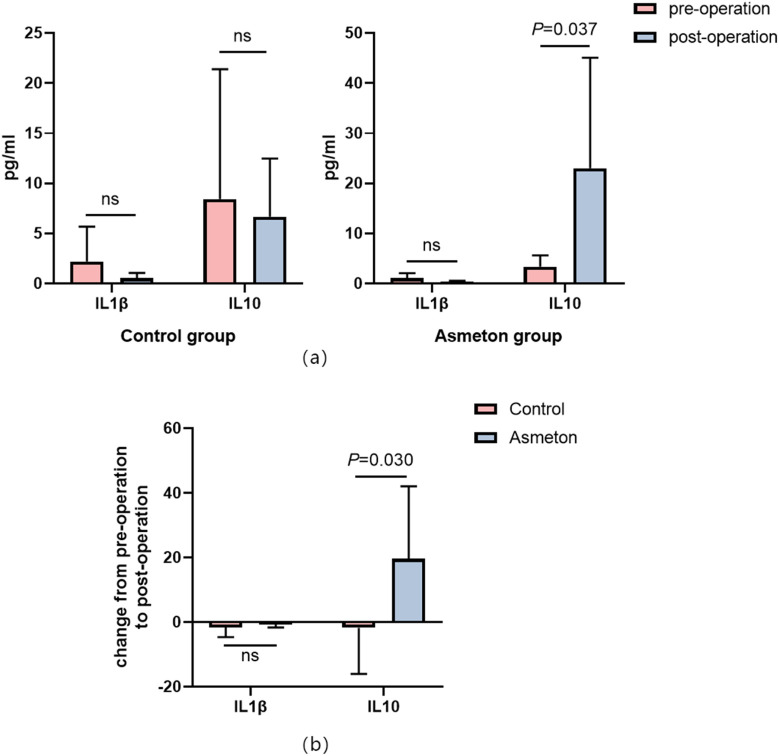
Changes in IL-1β and IL-10 with or without asmeton before and after operation. **(a)** Expression levels of IL-1β and IL-10 in the serum of the control and the asmeton groups. **(b)** Changes in IL-1β and IL-10 levels from pre-operation to post-operation.

We measured serum IL1β and IL10 levels of patients before and after operation. It was found that in both the control and asmeton groups, there was no significant difference in the IL1β levels before and after operation. However, asmeton might significantly increase the IL10 level in postoperative patients compared with the control group (*P* = 0.03) (Figure 5).

## Discussion

This study focused on PFA in 16 patients, exploring various aspects including patient baseline characteristics, procedural outcomes, ablation details, and the impact on diaphragm contraction and dry cough. We found that all patients could tolerate discomforts such as chest pain and headache during PFA under conscious sedation and local anesthesia. Dry cough and diaphragmatic contraction were unavoidable, but the use of asmeton can significantly reduce the degree of dry cough and diaphragmatic contraction, especially the proportion of moderate-to-severe cough.

Compared with traditional thermal ablation, PFA may be safer and cause fewer complications (Maor et al., 2019; Sugrue et al., 2019; Qiu et al., 2022; Reddy et al., 2018; Turagam et al., 2023; Reddy et al., 2023). However pulses are thought to cause underlying skeletal muscle contraction, so PFA is often performed under general anesthesia. PFA performed only under conscious sedation can result in increased diaphragm contraction and dry cough (Reddy et al., 2019; Reinsch et al., 2022). These problems cause patient discomfort, cause the patient’s body to move, or cause the 3D modeling to shift, which affects the stability of the intracardiac catheter and the continuity of the ablation sites, interrupts surgery, reduces the efficiency of PFA, and may even lead to a highly increased risk of heart rupture (Calvert et al., 2024b). In addition to general anesthesia, conscious sedation is also the most commonly used strategy in AF ablation surgery (Garcia et al., 2021). There has been fewer studies of PFA in patients with AF under conscious sedation, and the researchers do not recommend the use of conscious anesthesia during surgery in patients (Calvert et al., 2024b). Another study found that respiratory control under conscious sedation can minimize diaphragmatic contractions and dry coughs during AF PFA. However, performing respiratory control during the operation requires cooperation of the patient and the surgeon, increasing the difficulty of the operation (Jiang et al., 2023). Therefore, we need to find new methods to reduce the risk of diaphragmatic contractions and coughing in PFA under conscious anesthesia to promote the application of PFA in clinical practice and significantly reduce the risks associated with general anesthesia and the surgical preparation time.

Asmeton, also known as compound methoxylamine capsules, is a widely used drug in clinical practice. The main ingredients include aminophylline, methoxyphenamine, hydrochloride, noscapine, and chlorphenamine maleate. The drug plays an important role in the treatment of a variety of respiratory diseases. These four components can relieve dry cough through different mechanisms, such as relaxing bronchial smooth muscle, preventing the introduction of cough impulses, and reducing allergic reactions in the respiratory mucosa. Other studies have shown that asmeton can reduce cough response by reducing the levels of inflammatory indicators such as TNF-α, IL-1β, IL-6, and TGF-β (Wang et al., 2003). In this experiment, we found that asmeton did not affect IL-1β, but significantly increased the expression of the anti-inflammatory factor IL-10. One of the most likely causes for dry cough during PFA is direct stimulation of the bronchus near the ablation site. LSPV is closer to the bronchus than the RSPV, which explains why LSPV has a higher dry cough score than RSPV (LSPV 0.67 ± 0.59 vs. RSPV 0.07 ± 0.12). The right carina and RIPV did not have moderate-to-severe dry cough. The dry cough score was significantly reduced when PVI was performed with asmeton via PFA (Table 4), especially in LSPV, where dry cough was the most significant. Overall, asmeton eliminated nearly all moderate and severe incidences of dry coughs during PFA treatment of pulmonary veins (Figure 3).

In this study, phrenic nerve (PN) stimulation was not measured by objective means. The results were based on observations of diaphragmatic contraction. The right PN spanned the right atrium and the anterior inferior part of RSPV from above. Diaphragmatic contraction is finely regulated by the nervous system. Compared to the left pulmonary vein, the right pulmonary vein is more prone to diaphragmatic tremors, which may be related to the proximity of the right phrenic nerve to the right pulmonary vein. A published study found that the distance between the right PN and the RSPV was 1.4 ± 1.7 mm (Ichihara et al., 2016). For example, during PFA, the right pulmonary vein in the control group (score 0.77 ± 0.51, n = 83) was higher than the left pulmonary vein (score 0.32 ± 0.23, n = 83), which may explain why diaphragmatic contraction in the right pulmonary vein was stronger than that in the left pulmonary vein through PFA (Table 5). Asmeton may affect the body’s respiration to affect diaphragm contraction and can dilate the bronchial smooth muscle, improve ventilation function to reduce respiratory load, and thereby reduce diaphragm contraction (Liu et al., 2003). In contrast, aminophylline, a component of asmeton, can improve the contractility of the diaphragm by stimulating the PN, enhancing its resistance to fatigue (Aubier et al., 1981; Chiang et al., 1995), making the respiratory muscle activities more regular and coordinated, and, to some extent, helping correct possible respiratory disorders and reduce abnormal diaphragm contraction. Therefore, the decrease in the proportion of patients with a high degree of diaphragmatic constriction further supports the potential benefit of asmeton in reducing diaphragm-related complications (Figure 4).

This study has several limitations. The small sample size of 16 patients in the main ablation study may limit the generalizability of the findings. Additionally, the short-term follow-up period of 7 days for acute adverse events does not provide information on long-term outcomes, such as the recurrence of atrial fibrillation, late-onset complications, and the durability of PVI. Furthermore, the conclusions of this study are influenced by specific clinical and device-related contexts: The conscious sedation protocol employed differs significantly from the deep sedation or general anesthesia commonly used in other settings. While this represents the specific clinical scenario addressed, it necessitates caution when extrapolating the results to centers using deeper anesthesia protocols. The observed incidence of reactions, such as cough, may not be directly generalizable to other commercially available PFA systems as the specific catheter used (whose CE-mark application is actively underway) has unique pulse parameters and electric field profiles. In addition, the placement of the catheter in the pulmonary vein may affect the score. The orifice of the pulmonary vein serves as the main discharge site during the ablation procedure. If there are residual potentials inside the pulmonary vein, consolidation ablation will be carried out, and patients may respond more strongly to PFA. In this study, we did not record the depth of the ring electrode in the pulmonary vein or the specific number of electrodes during the ablation process, which may affect the analysis of the results. At the same time, the scoring method used is relatively subjective. To ensure data consistency and minimize the influence of subjective factors, all scoring was performed by the same technician. In addition, this study did not measure PN stimulation by objective means, which can be refined in future experiments to enroll larger and more diverse patient populations and conduct long-term follow-up. Ultimately, research efforts should aim to identify the predictors of PFA-induced cough and develop risk-stratification models to guide personalized prophylactic strategies.

## Conclusion

In conclusion, this study proposes a novel protocol, using asmeton only before surgery, which can effectively reduce the severity of diaphragmatic contraction and dry cough symptoms in AF patients through PFA under conscious sedation. The possible mechanism is that asmeton can promote anti-inflammatory responses to relieve cough and, at the same time, reduce abnormal diaphragmatic contractions by stimulating the PN.

## Data Availability

The raw data supporting the conclusions of this article will be made available by the authors, without undue reservation.

## References

[B1] Aubier M. De Troyer A. Sampson M. Macklem P. T. Roussos C. (1981). Aminophylline improves diaphragmatic contractility. N. Engl. J. Med. 305 (5), 249–252. 10.1056/NEJM198107303050503 7242614

[B2] Calkins H. Hindricks G. Cappato R. Kim Y. H. Saad E. B. Aguinaga L. (2017). 2017 HRS/EHRA/ECAS/APHRS/SOLAECE expert consensus statement on catheter and surgical ablation of atrial fibrillation. Heart Rhythm 14 (10), e275–e444. 10.1016/j.hrthm.2017.05.012 28506916 PMC6019327

[B3] Calvert P. Koniari I. Mills M. T. Ashrafi R. Snowdon R. Gupta D. (2024a). Lesion metrics and 12-month outcomes of very-high power short duration radiofrequency ablation (90W/4 s) under mild conscious sedation. J. Cardiovasc. Electrophysiol. 35 (6), 1165–1173. 10.1111/jce.16269 38571287

[B4] Calvert P. Mills M. T. Murray B. Kendall J. Ratnasingham J. Luther V. (2024b). Feasibility of pulsed field ablation for atrial fibrillation under mild conscious sedation. J. Interv. Card. Electrophysiol. 68, 1429–1436. 10.1007/s10840-024-01961-1 39623098 PMC12436502

[B5] Chiang C. H. Tang Y. C. Wang S. E. Hwang J. C. (1995). Changes in phrenic, hypoglossal and recurrent laryngeal nerve activities after intravenous infusions of aminophylline in cats. Eur. Respir. J. 8 (4), 632–636. 10.1183/09031936.95.08040632 7664865

[B6] Chu G. Calvert P. Sidhu B. Mavilakandy A. Kotb A. Tovmassian L. (2023). Patient experience of very high power short duration radiofrequency ablation for atrial fibrillation under mild conscious sedation. J. Interv. Card. Electrophysiol. 66 (2), 445–453. 10.1007/s10840-022-01351-5 35997848 PMC9396586

[B7] Ekanem E. Reddy V. Y. Schmidt B. Reichlin T. Neven K. Metzner A. (2022). Multi-national survey on the methods, efficacy, and safety on the post-approval clinical use of pulsed field ablation (MANIFEST-PF). Europace 24 (8), 1256–1266. 10.1093/europace/euac050 35647644 PMC9435639

[B8] Garcia R. Waldmann V. Vanduynhoven P. Nesti M. Jansen de Oliveira Figueiredo M. Narayanan K. (2021). Worldwide sedation strategies for atrial fibrillation ablation: current status and evolution over the last decade. Europace 23 (12), 2039–2045. 10.1093/europace/euab154 34308973

[B9] Ichihara N. Miyazaki S. Nakamura H. Taniguchi H. Takagi T. Hachiya H. (2016). Impact of catheter contact force on superior vena cava mapping and localization of the right phrenic nerve by high output pacing. J. Cardiovasc. Electrophysiol. 27 (3), 290–295. 10.1111/jce.12868 26511613

[B10] Jiang R. Liu Q. Chen L. Chen S. Wang Y. Cheng H. (2023). Respiratory control minimizes diaphragmatic contraction and dry cough during pulsed-field ablation of atrial fibrillation. Europace 26 (1), euad374. 10.1093/europace/euad374 38165731 PMC10781438

[B11] Liu X. S. Xu Y. J. Zhang Z. X. Li C. Q. Yang D. L. Zhang N. (2003). Isoprenaline and aminophylline relax bronchial smooth muscle by cAMP-induced stimulation of large-conductance Ca2+-activated K+ channels. Acta Pharmacol. Sin. 24 (5), 408–414. 12740175

[B12] Maor E. Sugrue A. Witt C. Vaidya V. R. DeSimone C. V. Asirvatham S. J. (2019). Pulsed electric fields for cardiac ablation and beyond: a state-of-the-art review. Heart Rhythm 16 (7), 1112–1120. 10.1016/j.hrthm.2019.01.012 30641148

[B13] Qiu J. Lan L. Wang Y. (2022). Pulsed electrical field in arrhythmia treatment: current status and future directions. Pacing Clin. Electrophysiol. 45 (10), 1255–1262. 10.1111/pace.14586 36029174

[B14] Reddy V. Y. Koruth J. Jais P. Petru J. Timko F. Skalsky I. (2018). Ablation of atrial fibrillation with pulsed electric fields: an ultra-rapid, tissue-selective modality for cardiac ablation. JACC Clin. Electrophysiol. 4 (8), 987–995. 10.1016/j.jacep.2018.04.005 30139499

[B15] Reddy V. Y. Neuzil P. Koruth J. S. Petru J. Funosako M. Cochet H. (2019). Pulsed field ablation for pulmonary vein isolation in atrial fibrillation. J. Am. Coll. Cardiol. 74 (3), 315–326. 10.1016/j.jacc.2019.04.021 31085321

[B16] Reddy V. Y. Gerstenfeld E. P. Natale A. Whang W. Cuoco F. A. Patel C. (2023). Pulsed field or conventional thermal ablation for paroxysmal atrial fibrillation. N. Engl. J. Med. 389 (18), 1660–1671. 10.1056/NEJMoa2307291 37634148

[B17] Reinsch N. Füting A. Höwel D. Bell J. Lin Y. Neven K. (2022). Cerebral safety after pulsed field ablation for paroxysmal atrial fibrillation. Heart Rhythm 19 (11), 1813–1818. 10.1016/j.hrthm.2022.06.018 35718318

[B18] Schmidt B. Bordignon S. Tohoku S. Chen S. Bologna F. Urbanek L. (2022). 5S study: safe and simple single shot pulmonary vein isolation with pulsed field ablation using sedation. Circ. Arrhythm. Electrophysiol. 15 (6), e010817. 10.1161/CIRCEP.121.010817 35617232

[B19] Sugrue A. Vaidya V. Witt C. DeSimone C. V. Yasin O. Maor E. (2019). Irreversible electroporation for catheter-based cardiac ablation: a systematic review of the preclinical experience. J. Interv. Card. Electrophysiol. 55 (3), 251–265. 10.1007/s10840-019-00574-3 31270656

[B20] Turagam M. K. Neuzil P. Schmidt B. Reichlin T. Neven K. Metzner A. (2023). Safety and effectiveness of pulsed field ablation to treat atrial fibrillation: one-year outcomes from the MANIFEST-PF registry. Circulation 148 (1), 35–46. 10.1161/CIRCULATIONAHA.123.064959 37199171

[B21] Wang Y. H. Bai C. X. Hong Q. Y. Chen J. (2003). Anti-inflammatory effect of methoxyphenamine compound in rat model of chronic obstructive pulmonary disease. Acta Pharmacol. Sin. 24 (12), 1324–1327. 14653967

