# Supplementary material for: Asmeton minimizes dry cough and diaphragmatic contraction during pulsed-field ablation of atrial fibrillation: a clinical prospective randomized study
Source: Front Physiol. 2026 Feb 18;16:1702254. doi: 10.3389/fphys.2025.1702254 (PMC12956715; doi:10.3389/fphys.2025.1702254)
Supplement: Supplementary file 1 [file Table1.DOCX]

**Table Supplement 1 Intraoperative Feeling Scale**

| Score | Description |
| --- | --- |
| 0 | No discomfort |
| 1 | The symptoms are mild and slightly uncomfortable |
| 2 | The symptoms are obvious, but they are tolerable |
| 3 | The symptoms are severe, but there is no need to suspend the ablation |
| 4 | The symptoms are very severe, feeling unable to breathe, requiring a pause in ablation |

**Table Supplement 2 Rating chart of pulsed-field ablation impact on dry cough**

| Degree | Score | Description |
| --- | --- | --- |
| None | 0 | No or neglectable dry cough |
| Low | 1 | Mild dry cough (slight but noticeable scattering cough) |
| Medium | 2 | Moderate dry cough (obvious but scattering cough) |
| High | 3 | Severe dry cough (loud and continuous cough) |

**Table Supplement 3 Rating chart of pulsed-field ablation impact on diaphragm contraction**

| Degree | Score | Description |
| --- | --- | --- |
| None | 0 | No or neglectable muscle contraction |
| Low | 1 | Mild diaphragm contraction (slight but noticeable abdomen movement) |
| Medium | 2 | Moderate diaphragm contraction (obvious abdomen movement) |
| High | 3 | Severe diaphragm contraction (strong abdomen movement or slight body bounce) |
